# Supplementary material for: Association of Differentially Altered Liver Fibrosis with Deposition of TGFBi in Stabilin-Deficient Mice
Source: Int J Mol Sci. 2023 Jun 30;24(13):10969. doi: 10.3390/ijms241310969 (PMC10341388; doi:10.3390/ijms241310969)
Supplement: Supplementary file 1 [file ijms-24-10969-s001.zip › ijms-2440967-supplementary.docx]

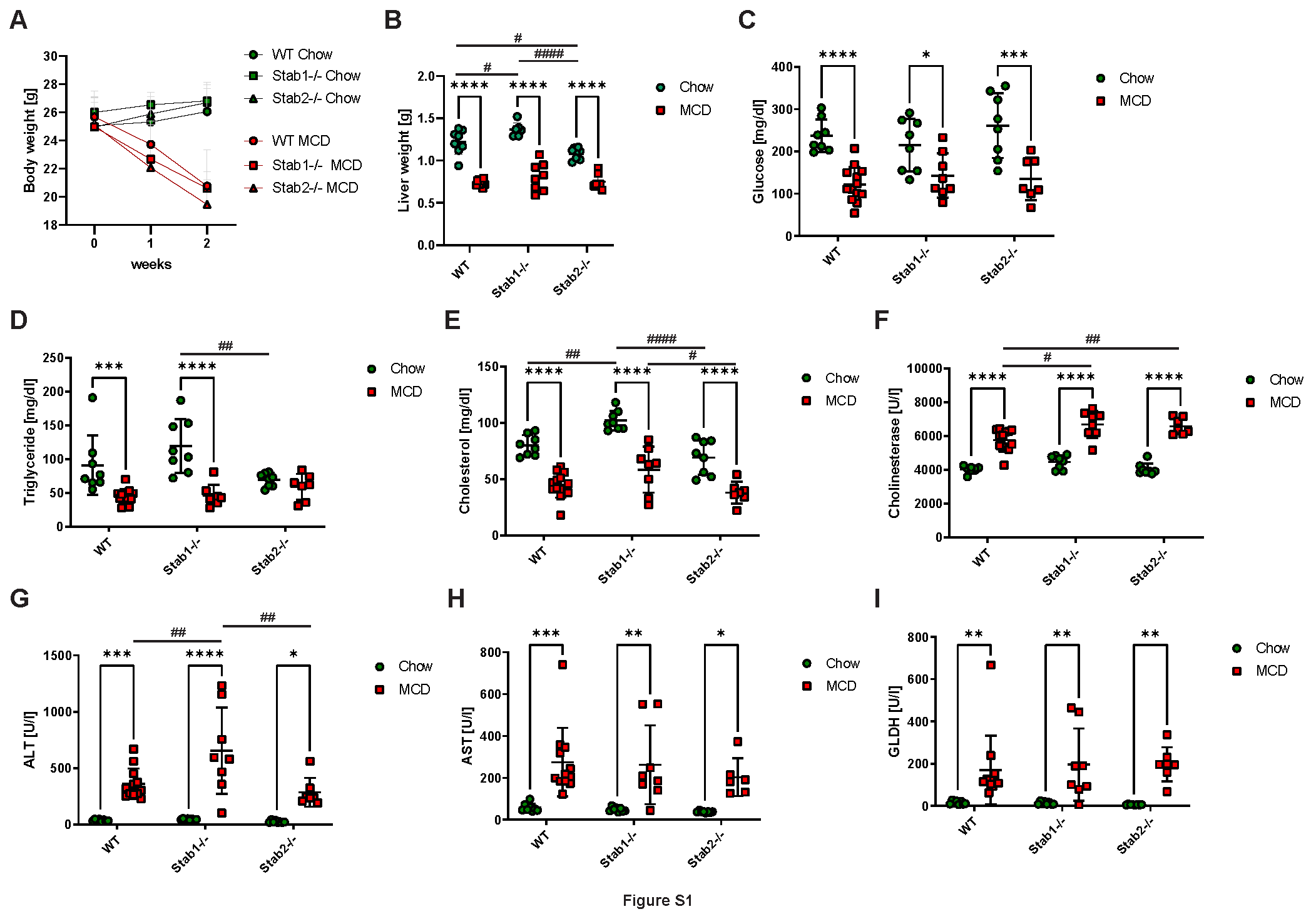


**Figure S1: Metabolic profile and liver enzymes of Chow and MCD fed mice.** (A) Body weight progression during Chow and MCD diet. (B) Liver / Body weight Ratio of Chow and MCD mice. Plasma levels of (C) Glucose, (D) Triglycerides, (E) Cholesterol (F) Cholinesterase, (G) ALT, (H) AST and (I) GLDH. * show significances between treatments and # show significances between genotypes. The symbols show the following significances: *p* ≤ 0.05 = */#; *p* < 0.01 = **/##; *p* < 0.001 = ***; *p* < 0.0001 = ****/####.


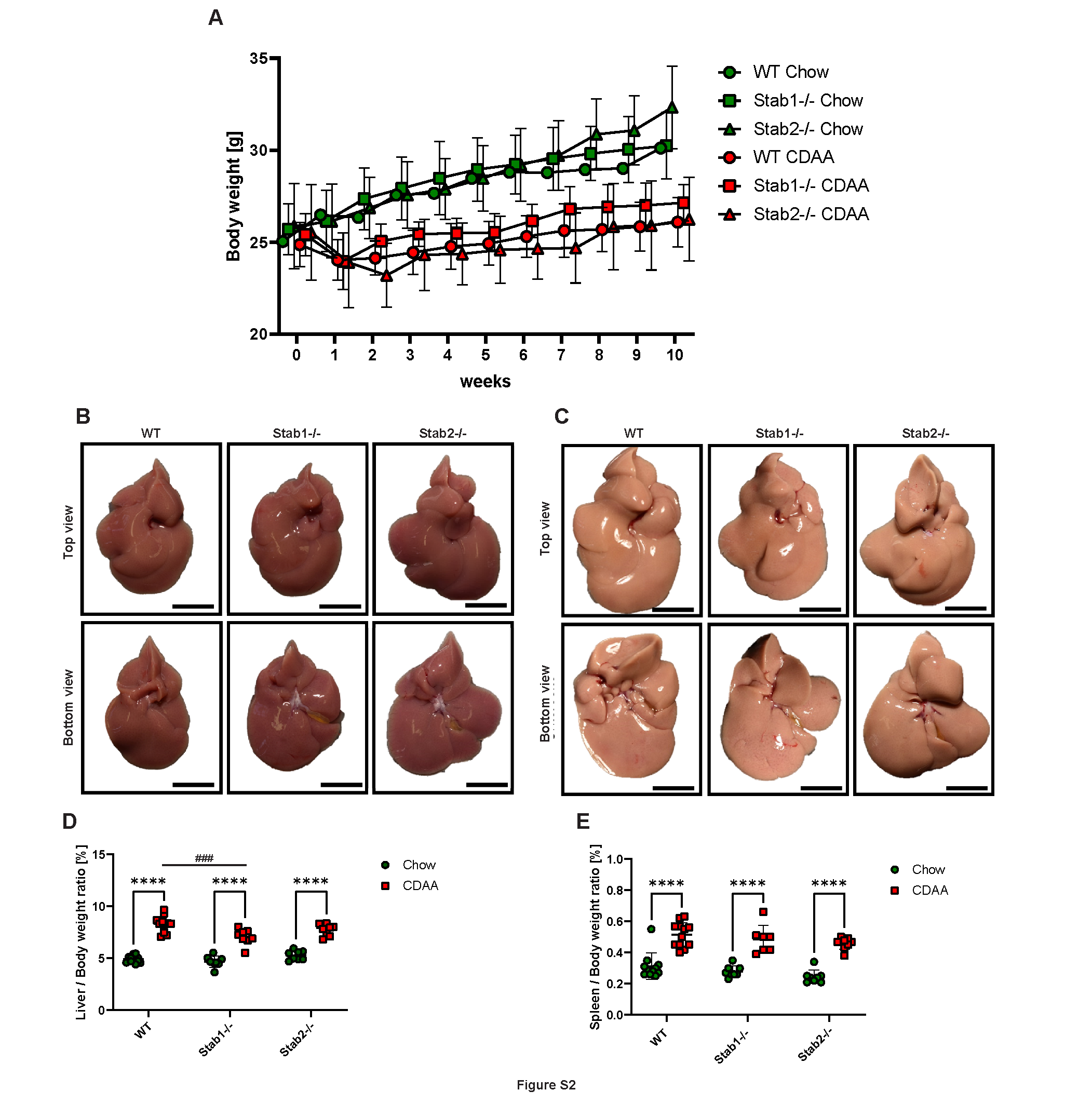


**Figure S2: Effects of CDAA diet on physiology.** (A) Body weight progression during Chow and CDAA diet. (B) Examples of livers from Chow fed mice (scale bar = 1 cm). (C) Examples of livers from CDAA fed mice (scale bar = 1 cm). (D) Liver / Body weight Ratio of Chow and CDAA mice. (E) Spleen / Body weight Ratio of Chow and CDAA mice. * show significances between treatments and # show significances between genotypes. The symbols show the following significances: *p* < 0.001 = ###; *p* < 0.0001 = ****.


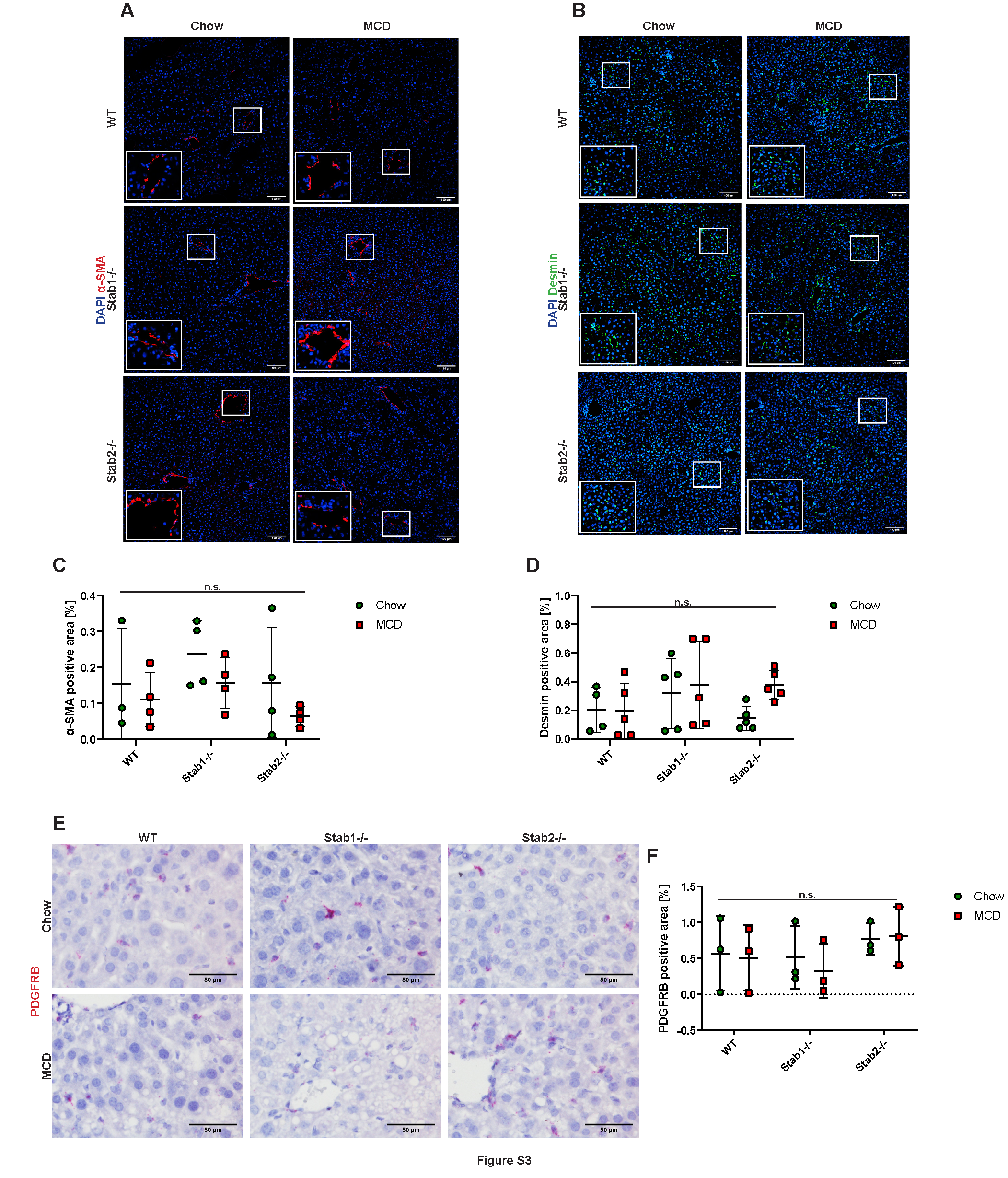


**Figure S3: Effects of MCD diet on hepatic stellate cells.** (A) α-SMA IF staining of representative liver sections (scale bar = 100 µm). (B) Desmin IF staining of representative liver sections (scale bar = 100 µm). (C) Quantification of α-SMA positive staining. (D) Quantification of Desmin positive staining. (E) PDGFRB In Situ Hybridization of representative liver sections (scale bar = 50 µm). (F) Quantification of PDGFRB positive staining. n.s. means not significant.


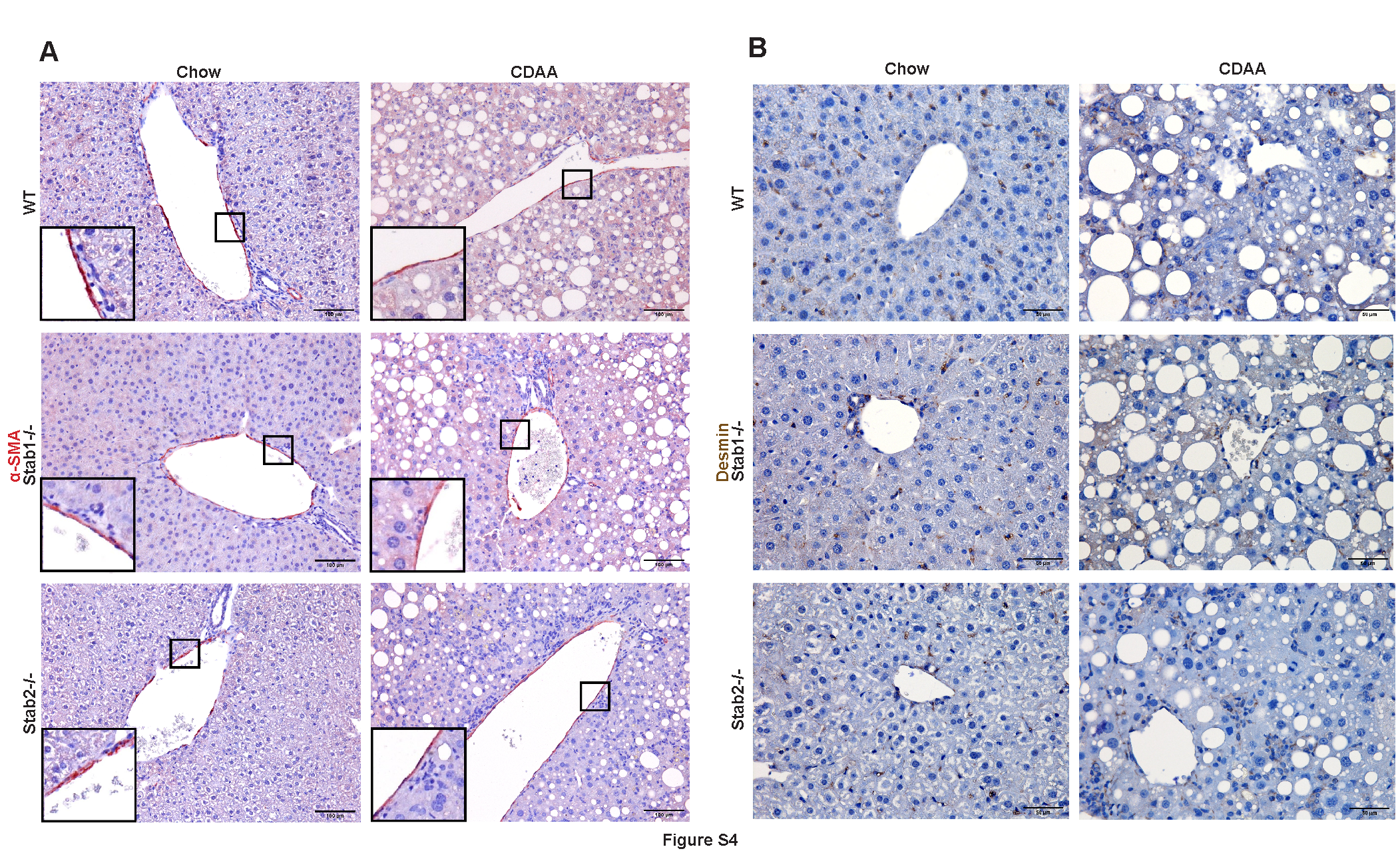


**Figure S4: Effects of CDAA diet on hepatic stellate cells.** (A) α-SMA IF staining of representative liver sections (scale bar = 100 µm). (B) Desmin IF staining of representative liver sections (scale bar = 50 µm).


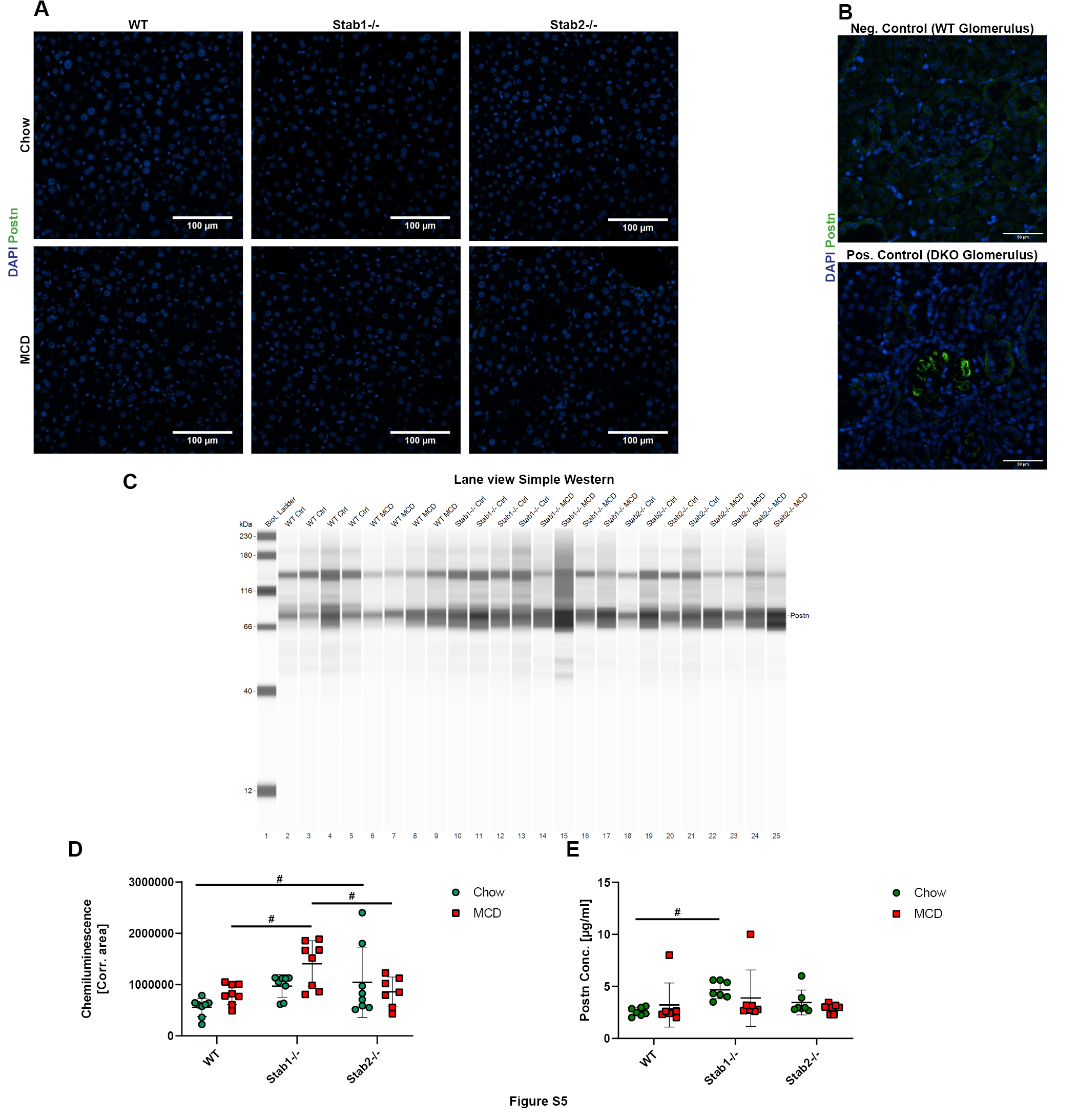


**Figure S5: Effects of MCD diet on stabilin ligand POSTN.** (A) POSTN IF staining of representative liver sections (scale bar = 100 µm). (B) POSTN IF staining of DKO glomerulus as a positive control (scale bar = 50 µm). (C) Lane view of POSTN Simple Western from liver protein. (D) Quantification of POSTN Simple Western. (E) Quantification of POSTN ELISA from Plasma. # show significances between genotypes. The symbols show the following significances: *p* ≤ 0.05 = #.


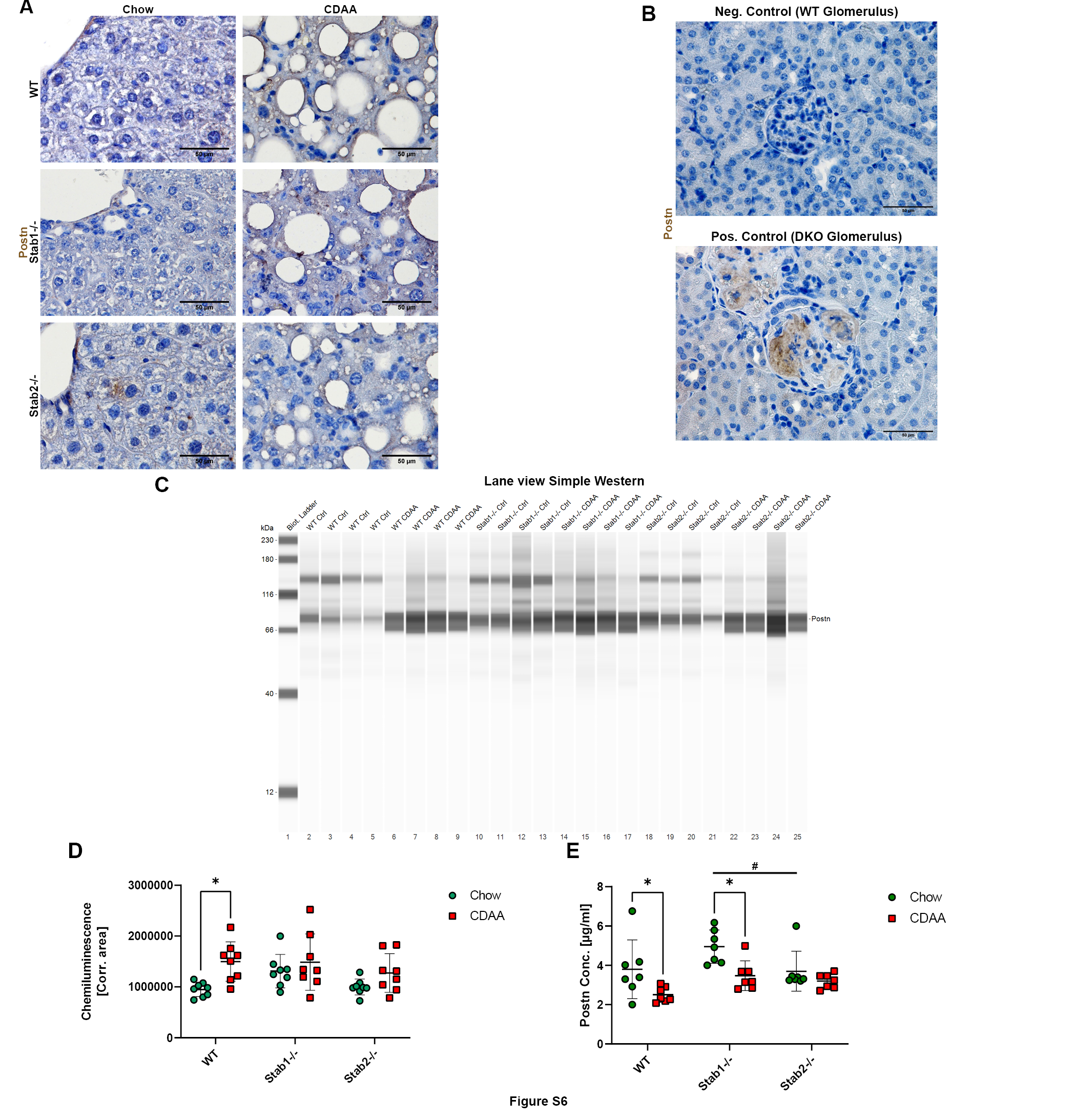


**Figure S6: Effects of CDAA diet on stabilin ligand POSTN.** (A) POSTN IHC staining of representative liver sections (scale bar = 100 µm). (B) POSTN IHC staining of DKO glomerulus as a positive control (scale bar = 50 µm). (C) Lane view of POSTN Simple Western from liver protein. (D) Quantification of POSTN Simple Western. (E) Quantification of POSTN ELISA from Plasma. * show significances between treatments. The symbols show the following significances: *p* ≤ 0.05 = *.

**Abbreviation list**

| **Abbreviation** | **Full text** |
| --- | --- |
| LSECs | Liver sinusoidal endothelial cells |
| TGFBi | Transforming growth factor, beta-induced, 68kDa |
| POSTN | Periostin |
| Stab1 | Stabilin-1 |
| Stab2 | Stabilin-2 |
| WT | Wildtype |
| MCD | Methionine-Choline-deficient |
| CDAA | Choline-deficient L-amino acid-defined |
| MS-1 | MS-1 high-molecular weight protein |
| HA | Hyaluronan |
| Stab1-deficient | *Stab1-/-* |
| Stab2-deficient | *Stab2-/-* |
| Stab-DKO | Stab1/2-double-deficient |
| NASH | nonalcoholic steatohepatitis |
| PDGFRB | Platelet-derived growth factor receptor beta |
| Emcn | Endomucin |
| ICAM-1 | Intercellular Adhesion Molecule 1 |
| IF | Immunofluorescent |
